# The prognostic and predictive potential of Ki-67 in triple-negative breast cancer

**DOI:** 10.1038/s41598-019-57094-3

**Published:** 2020-01-14

**Authors:** Xiuzhi Zhu, Li Chen, Binhao Huang, Yue Wang, Lei Ji, Jiong Wu, Genhong Di, Guangyu Liu, Keda Yu, Zhimin Shao, Zhonghua Wang

**Affiliations:** 1 Department of Oncology, Shanghai Medical College, Fudan University, 130 Dong-An Road, Shanghai, 200032 P.R. China; 2 Key Laboratory of Breast Cancer in Shanghai, Fudan University Shanghai Cancer Center, 270 Dong-An Road, Shanghai, 200032 P.R. China; 3 Department of Breast Surgery, Fudan University Shanghai Cancer Center, 270 Dong-An Road, Shanghai, 200032 P.R. China; 4 Department of Gastric Surgery, Fudan University Shanghai Cancer Center, 270 Dong-An Road, Shanghai, 200032 P.R. China; 5 Department of Pathology, Fudan University Shanghai Cancer Center, 270 Dong-An Road, Shanghai, 200032 P.R. China; 6 Institutes of Biomedical Sciences, Fudan University, Shanghai, P.R. China

**Keywords:** Breast cancer, Tumour biomarkers, Breast cancer

## Abstract

As a cell proliferation biomarker, Ki-67 is principally used in ER+/HER2− breast cancer. However, the importance and the best cutoff point of Ki-67 in triple-negative breast cancer (TNBC) remains unclear and was evaluated in this study.A total of 1800 patients with early invasive TNBC between 2011 and 2016 at Fudan University Shanghai Cancer Center were consecutively recruited for this study. The optimal cutoff for Ki-67 was assessed by Cutoff Finder. Propensity score matching (PSM, ratio = 1:2) was performed to match the Ki-67^low^ group with the Ki-67^high^ group. Overall survival (OS) and disease-free survival (DFS) were compared between the two groups using the Kaplan-Meier method and Cox regression model. The most relevant cutoff value for Ki-67 for prognosis was 30% (*p* = 0.008). At the cutoff point of 30%, worse DFS and OS were observed in the Ki-67^high^ group. In multivariate analyses, N-stage (*p* < 0.001), T-stage (*p* = 0.038), and Ki-67 at the 30% threshold (*p* = 0.020) were independently linked to OS. In subgroup analysis, Ki-67 cutoff at 30% had prognostic and predictive potential for DFS with either tumor size ≤2 cm (*p* = 0.008) or lymph node-negative (N−) (*p* = 0.038) and especially with T_1_N_0_M_0_ (stage I) TNBCs. For 945 N− TNBC patients, adjuvant chemotherapy (CT) was associated with better OS in the Ki-67^high^ group (*p* = 0.017) than in the Ki-67^low^ group (*p* = 0.875). For stage I/Ki-67^low^ patients, adjuvant CT did not affect DFS (*p* = 0.248). Thus, Ki-67 cutoff at 30% had early independent prognostic and predictive potential for OS and DFS in TNBCs, and Ki-67 > 30% was significantly associated with worse prognosis, especially for stage I patients. For stage I/Ki-67^low^ TNBC patients, the advantage of CT is unclear, providing the basis for future de-escalation therapy.

## Introduction

Triple-negative breast cancer (TNBC) accounts for approximately 15% of all breast cancers (BCs) and lacks estrogen receptor (ER) and progesterone receptor (PR) expression as well as human epidermal growth factor receptor 2 (HER2) amplification. Compared to other BC subtypes, patients with TNBC currently cannot benefit from targeted therapy or endocrine therapy. A higher recurrence rate and a higher frequency of metastasis are often observed in more biologically aggressive TNBCs in contrast with other subtypes. Therefore, TNBC has poor prognosis and worse survival^1,2^.

The expression of biomarkers is one of the important factors for treatment strategy decision-making. The prognostic factors for BC include histological features (histological type, histological grade, and lymphovascular invasion), lymph node status, tumor size, steroid hormone receptor status and age^3^. Recently, a new panel of biomarkers has been identified to provide both prcognostic and predictive information for BC. Among these new biomarkers, Ki-67 is one of the most promising markers. Ki-67 is a protein expressed in the nucleus of cells during different phases of the cell cycle, except in the G0 quiescent state. As one of the most widely used immunohistochemistry (IHC) proliferation antigens, Ki-67 has been proposed to be a measure for the quantification of cell proliferation in BC samples^4^. In general, high levels of Ki-67 expression in BC correlate strongly with more tenacious proliferation and poor prognosis. In a study conducted by Cheang^5^, the cutoff value for the immunohistochemically determined Ki-67 index to distinguish luminal B from luminal A BC was 13.25%, while some panels showed that a threshold of ≥20% was clearly indicative of ‘high’ Ki-67 status^6^. Currently, Ki-67 is principally used to evaluate prognosis, guide adjuvant treatment and predict the response to neoadjuvant treatment in ER+/HER2− BC. The Ki-67 cutoff at 14% is an important parameter in subclassifying luminal BC into the luminal A subtype with good prognosis and the luminal B subtype with worse prognosis^6–8^.

However, the importance and the best cutoff point of this marker as a prognostic and predictive factor in TNBC remains unclear. In the current study, we evaluated the prognostic and predictive potential of Ki-67 in TNBC.

## Materials and Methods

### Patients

**Figure 1:**
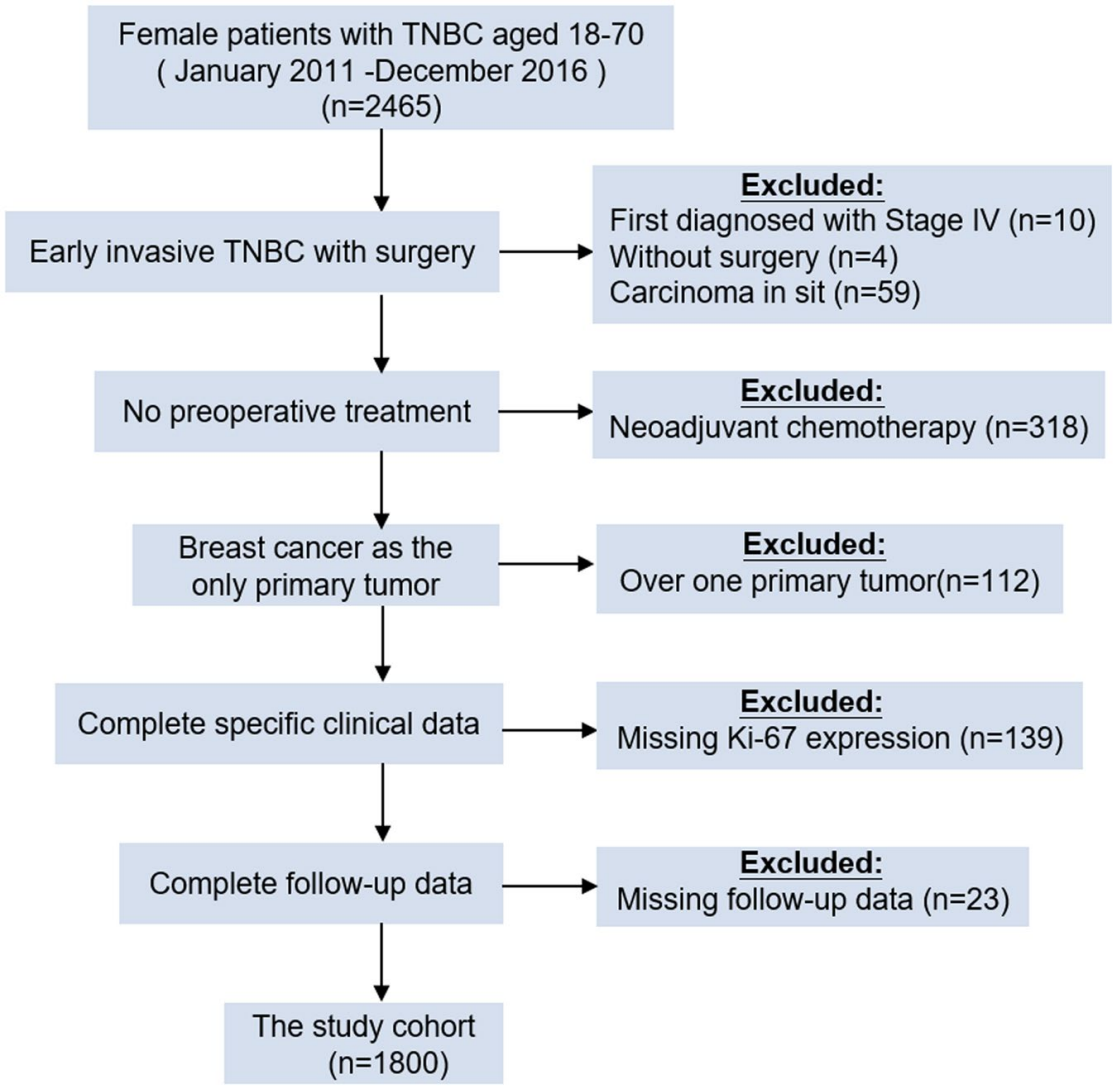
Flowchart of inclusion and exclusion criteria for the study. 1800 cases were enrolled. Abbreviations: TNBC, triple-negative breast cancer.

A total of 2465 female patients with TNBC between January 2011 and December 2016 at Fudan University Shanghai Cancer Center (FUSCC) were consecutively recruited, as Ki-67 was not widely used in FUSCC as a routine test until 2011. After screening, only 1800 patients who were diagnosed with early invasive TNBC and underwent surgery were enrolled for this study. The other inclusion criteria for all participants were 18–70 years of age and BC as the only primary tumor. The exclusion criteria were neoadjuvant chemotherapy (CT) or radiotherapy (RT), deficiency of clinical data or lack of follow-up (Fig. 1).

Written permission was obtained for the collection of data from the FUSCC database, allowing the use of personal data for research purposes. The study was ethically approved by the Ethical Committee and Institutional Review Board of FUSCC, and all the methods were performed in accordance with the approved guidelines.

### Pathology and immunohistochemistry

The ER, PR, and HER2 status and Ki-67 expression of paraffin-embedded tissue sections of breast surgical tumor specimens from the pathology center of FUSCC were evaluated according to standard procedures. All surgical specimens were fixed with 10% neutral phosphate-buffered formalin and paraffin-embedded 4 μm-thick slices of representative tumor blocks were stained with hematoxylin and eosin. The cutoff values for ER/PR positivity were 1% of positive tumor cells with nuclear staining by IHC. HER2 positivity was defined as 3+ by IHC using circumferential membrane-bound staining or as positive on fluorescence *in situ* hybridization (FISH), whereas HER2 negativity was defined as 0 to 1+ by IHC or 2+ by IHC without gene amplification confirmed by FISH detection^9^. TNBC was defined only when the tumors were ER−, PR− and HER2-negative.

The immunohistochemical analysis for Ki-67 value was performed on Benchmark XT system (Ventana, Tucson, AZ, USA), using MIB-1 antibody (dilution 1:100; Code M7240, Dako, Glostrup, Denmark). The reported Ki-67 index was their average and reported as the percentage of nuclear staining-positive cells (at least 1000). The percentage of positive staining was evaluated by two independent pathologists.

### Clinical outcome assessment

Overall survival (OS) was calculated as the time from the initial pathological diagnosis to death from any cause. Disease-free survival (DFS) was defined as the period from the initial pathological diagnosis to recurrence, metastasis or BC-related death. All patients were followed-up to the date of death or March 12, 2019, which was the date of the last censorship and termination of the study. Patients with missing follow-up data were excluded from the analyses.

### Statistical analysis

The optimal cutoff for Ki-67 was assessed by Cutoff Finder^10^, which is an online software used for the analysis of important prognostic cutoff values (Charite, Berlin, http://molpath.charite.de/cutoff/). We defined patients with Ki-67 expression above the cutoff point as the Ki-67^high^ group, otherwise as the Ki-67^low^ group. Propensity score matching^11^ (PSM, ratio = 1:2) was performed to match the patients in the Ki-67^low^ group with those in the Ki-67^high^ group to balance baseline characteristics and potential prognostic confounders^12–14^, including age, BMI, location, T-stage, multifocality and N-stage. The chi-square test for categorical variables was used for comparisons between groups. OS and DFS were compared between the two groups using the Kaplan-Meier method and a Cox regression model. A *p* value < 0.05 was considered to be statistically significant. SPSS version 22.0 software (SPSS, Chicago, IL) and R software version 3.5.3. (The R Project for Statistical Computing, https://www.r-project.org/) were used for the calculations and analyses. The R packages “MatchIt”, “survminer”, “readr”, “survival”, and “forestplot” with the appropriate libraries were used.

### Ethical approval

All procedures performed in studies involving human participants were in accordance with the ethical standards of the institutional and/or national research committee and with the 1964 Helsinki declaration and its later amendments or comparable ethical standards.

### Informed consent

Informed consent was obtained from all individual participants included in the study.

## Results

### Patient characteristics of the overall cohort

For the 1800 invasive TNBC patients enrolled in this study, the median follow-up time was 43.2 months. The patients’ median age and BMI were 50 years (range 18–70 years) and 22.76, respectively. Regarding the main pathological types in this study, invasive ductal carcinoma was found in 79.56% of patients, and invasive carcinoma with *in situ* components was found in 11.56%. Among all patients, 72.39% were grade III and17.28% were grade II. Besides, there were 67.89% (1222/1800) patients without lymph node metastasis and 45.61% (821/1800) with tumor size ≤2 cm. Based on TNM stage, 34.28% (617/1800) of patients had stage I, 43.56% (784/1800) of patients had stage II, and 10.94% (197/1800) of patients had stage III disease. The median Ki-67 value was 60%, while Ki-67 > 20% accounted for 84.39% of patients, which is similar to the findings of a previous study. In the overall cohort, 71.89% received mastectomy and 48.61% explicitly accepted axillary dissection. In terms of adjuvant therapy regimens, 85.22% received adjuvant CT and 39.22% had adjuvant RT. More patients got adjuvant therapy in the Ki-67^high^ group (adjuvant CT: 87.05% vs 79.96%; adjuvant RT: 41.92% vs 31.47%). Of the 1534 who had adjuvant CT, 69.03% were received anthracene in combination with taxa chemotherapy. Other related characteristics of the patients are shown in Table 1

**Table 1:** Characteristics of the 1800 triple-negative breast cancer patients.

Variable	Before PSM n = 1800		
	Ki-67^Low^ (n = 464)	^a^Ki-67^High^ (n = 1336)	Total
	Number (%)	Number (%)	
**Age (year)**		^**b**^***p*** **< 0.001**	
≤50	155 (33.41%)	739 (55.31%)	894 (49.67%)
>50	309 (66.59%)	597 (44.69%)	906 (50.33%)
**BMI**		***p*** = **0.4745**	
≤24	295 (63.58%)	870 (65.12%)	1165 (64.72%)
>24	161 (34.7%)	452 (33.83%)	613 (34.06%)
Missing	8 (1.72%)	14 (1.05%)	22 (1.22%)
**Location**		***p*** = **0.5283**	
Left	235 (50.65%)	652 (48.8%)	887 (49.28%)
Right	229 (49.35%)	684 (51.2%)	913 (50.72%)
**Multifocality**		***p*** = **0.4836**	
No	438 (94.4%)	1267 (94.84%)	1705 (94.72%)
Yes	18 (3.88%)	39 (2.92%)	57 (3.17%)
Missing	8 (1.72%)	30 (2.25%)	38 (2.11%)
**Differentiation**		***p*** **< 0.001**	
I	3 (0.65%)	0 (0%)	3 (0.17%)
II	168 (36.21%)	143 (10.7%)	311 (17.28%)
III	184 (39.66%)	1119 (83.76%)	1303 (72.39%)
Missing	109 (23.49%)	74 (5.54%)	183 (10.17%)
**pT**		***p*** = **0.018**	
T1	231 (49.78%)	590 (44.16%)	821 (45.61%)
T2	173 (37.28%)	566 (42.37%)	739 (41.06%)
T3	14 (3.02%)	20 (1.49%)	33 (1.89%)
Missing	46 (9.91%)	160 (11.98%)	206 (11.44%)
**pN**		***p*** = **0.1932**	
N0	325 (70.04%)	897 (67.14%)	1222 (67.89%)
N1	88 (18.97%)	298 (22.31%)	386 (21.44%)
N2	27 (5.82%)	92 (6.89%)	119 (6.61%)
N3	24 (5.17%)	49 (3.67%)	73 (4.06%)
**Surgery type**		***p*** **< 0.001**	
Mastectomy	370 (79.74%)	924 (69.16%)	1294 (71.89%)
Lumpectomy	94 (20.26%)	412 (30.84%)	506 (28.11%)
**Adjuvant chemotherapy**		***p*** **< 0.001**	
No	42 (9.05%)	33 (2.47%)	75 (4.17%)
Yes	371 (79.96%)	1163 (87.05%)	1534 (85.22%)
Missing	51 (10.99%)	140 (10.48%)	191 (10.61%)
**Adjuvant radiotherapy**		***p*** **< 0.001**	
No	264 (56.9%)	621 (46.48%)	885 (49.17%)
Yes	146 (31.47%)	560 (41.92%)	706 (39.22%)
Missing	54 (11.64%)	155 (11.6%)	209 (11.61%)

Abbreviations: PSM, propensity score matching.

^a^High Ki-67 expression was defined as >30%.

^b^*p* value was derived from the chi-square test

.

### Cutoff value for Ki-67 verified in the PSM cohort

**Figure 2:**
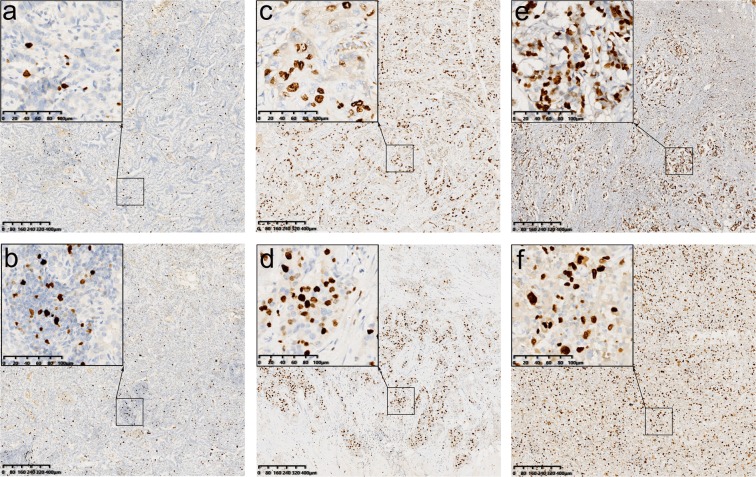
Immunohistochemical analysis of Ki-67 in six representative samples. The average score across the whole section should be taken. (**a**,**b**) Represent the Ki-67^low^ group; (**c**,**d**) represent the Ki-67 at the 30% threshold; (**e**,**f**) represent the Ki-67^high^ group. (**a**) The Ki-67 index was reported at 5%. (**b**) The Ki-67 index was reported at 10%. (**c**) The Ki-67 index was reported at 30%. (**d**) The Ki-67 index was reported at 30%. (**e**) The Ki-67 index was reported at 50%. (**f**) The Ki-67 index was reported at 60%. (MIB1 stain, scale bar = 80 µm and scale bar of inset = 20 µm).

To define the most relevant cutoff value for Ki-67 distinguishing prognosis, we used the online tool “Cutoff Finder”. This method has been employed by several other studies for the prognostic determination of various tumors^15–17^. The optimal cutoff value of Ki-67 for distinguishing DFS was 30% (p = 0.020). We defined patients with Ki-67 > 30% as the Ki-67^high^ group, otherwise as the Ki-67^low^ group. The representative IHC images of the Ki-67^high^ group, the Ki-67^low^ group and Ki-67 at the 30% threshold were presented in Fig. 2. There were 464 (74.2%) patients with Ki-67 ≤ 30% and 1336 (74.2%) with Ki-67 > 30%. To exclude differences in other baseline levels that might affect the prognosis of the two groups, PSM (ratio = 1:2) was performed to match the patients in the Ki-67^low^ group with those in the Ki-67^high^ group. After PSM, all baseline characteristics (age, BMI, location, multifocality, T-stage, and N-stage) of the two groups were well balanced (Table S1). There were 464 patients with Ki-67 ≤ 30% and 928 patients with Ki-67 > 30%, with 1392 patients total in the PSM cohort. At the cutoff point of 30%, worse DFS and OS were observed in the Ki-67^high^ group of the PSM cohort (*p* = *0.008 and p* = *0.043*, respectively).

### Ki-67 cutoff at 30% is an independent factor for OS and DFS

For the 1392 patients, Ki-67 > 30%, larger tumor size and lymph node positivity were associated with shorter DFS and OS in the combined PSM and univariate Cox regression analysis, while the other clinical characteristics, such as age, multifocality and histological subtype, did not influence the prognosis. In the combined PSM and multivariate Cox regression analysis, N-stage (*p* < 0.001), T-stage (*p* = 0.038), and Ki-67 at the 30% threshold (*p* = 0.020) were independently linked to OS. However, only N-stage (*p* < 0.001) and Ki-67 at the 30% threshold (*p* = 0.010) were independently linked to DFS. The hazard ratios (HRs) of the high expression of Ki-67 were 1.947 (95% CI: 1.108–3.421) for OS and 1.604 (95% CI: 1.118–2.300) for DFS, with the low-level of Ki-67 used as a reference. All the results from the univariate analysis and final multivariate Cox regression model are presented in Tables 2 and S2

**Table 2:** Univariate and multivariate Cox regression analyses of factors associated with overall survival in triple-negative breast cancer.

Variable		Univariate			Multivariate		
		HR	95% CI	*P* value	HR	95% CI	*P* value
Age	≤50 vs >50	1.508	0.935–2.434	0.092	1.529	0.887–2.636	0.127
BMI	≤24 vs >24	1.285	0.837–1.973	0.252			
Location	Left vs right	1.015	0.663–1.553	0.945			
Differentiation	III vs I + II	1.181	0.706–1.978	0.526			
**T-stage**				**0.000**			**0.038**
	pT2 vs pT1	2.924	1.827–4.680	0.000	1.947	1.167–3.251	0.011
	pT3 vs pT1	2.061	0.488–8.704	0.325	1.359	0.310–5.967	0.684
Multifocality	Yes vs no	2.144	0.864–5.323	0.100	1.830	0.733–4.569	0.196
**N-stage**				**0.000**			**0.000**
	pN1 vs pN0	2.451	1.408–4.265	0.002	2.168	1.183–3.972	0.012
	pN2vs pN0	5.071	2.774–9.271	0.000	3.980	1.983–7.988	0.000
	pN3 vs pN0	7.953	4.399–14.376	0.000	7.118	3.703–13.689	0.000
**Ki-67**	≤30% vs >30%	1.654	1.010–2.709	0.046	1.947	1.108–3.421	**0.020**

Abbreviations: CI, confidence interval; HR, hazard ratio.

The covariates in the Cox model were all categorical variables, and the adjusted *p* value and HR were derived from the model.

.

### Subgroup analysis: prognostic value of Ki-67 cutoff at 30% in the Cox regression of OS and DFS

**Figure 3:**
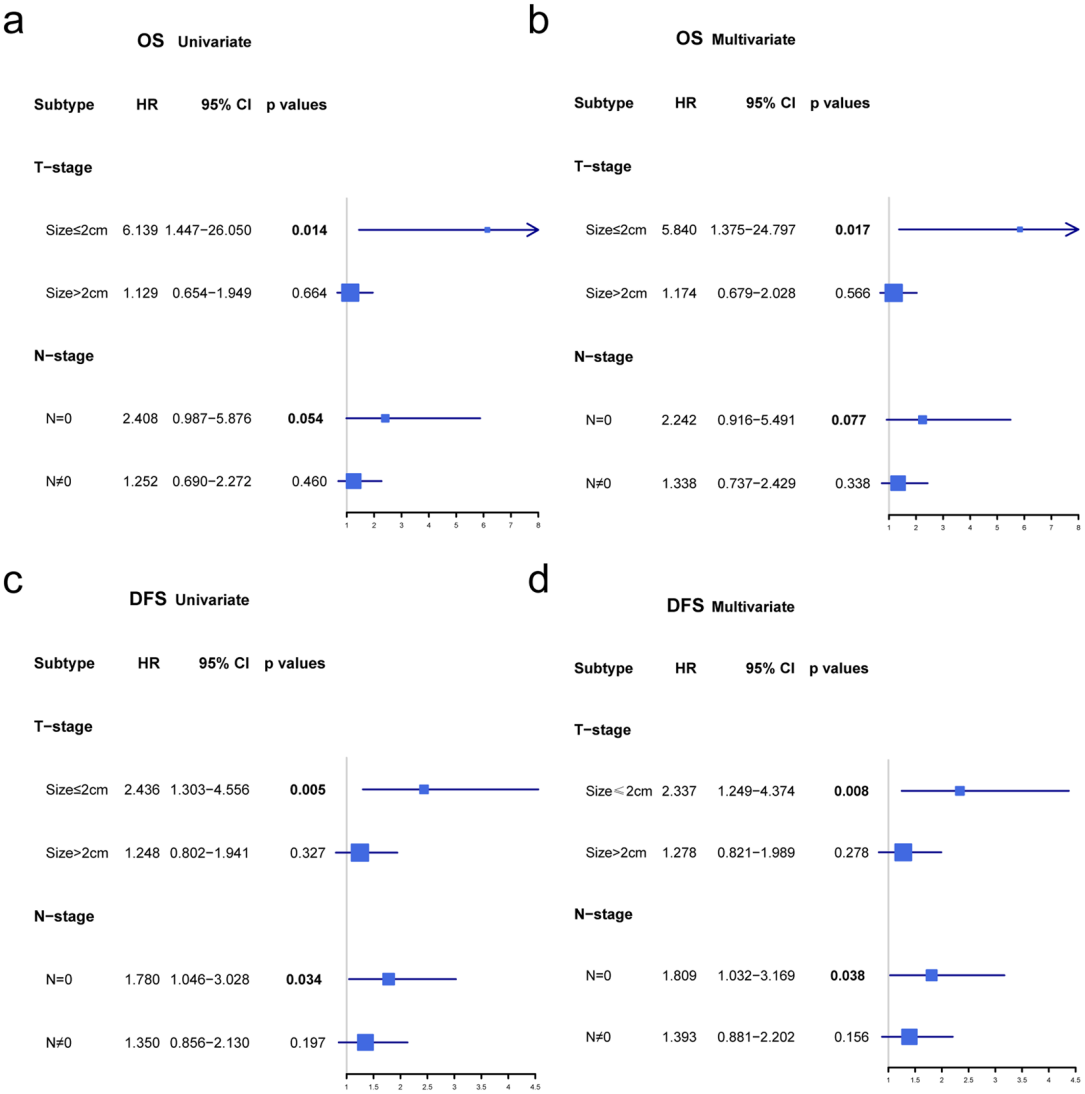
Subgroup analysis of the univariate and multivariate Cox regression models. It shows the difference in overall survival and disease-free survival between the Ki-67^low^ group and the Ki-67^high^ group. The covariates in the Cox model were all categorical variables, including Ki-67 and TNM. The adjusted *p* value and hazard ratio were derived from the Cox model, with Ki-67 ≤ 30% as a reference. (**a**) Univariate Cox regression model for overall survival; (**b**) multivariate Cox regression model for overall survival; (**c**) univariate Cox regression model for disease-free survival; (**d**) multivariate Cox regression model for disease-free survival. Abbreviations: CI, confidence interval; HR, hazard ratio; DFS, disease-free survival; OS, overall survival.

**Figure 4:**
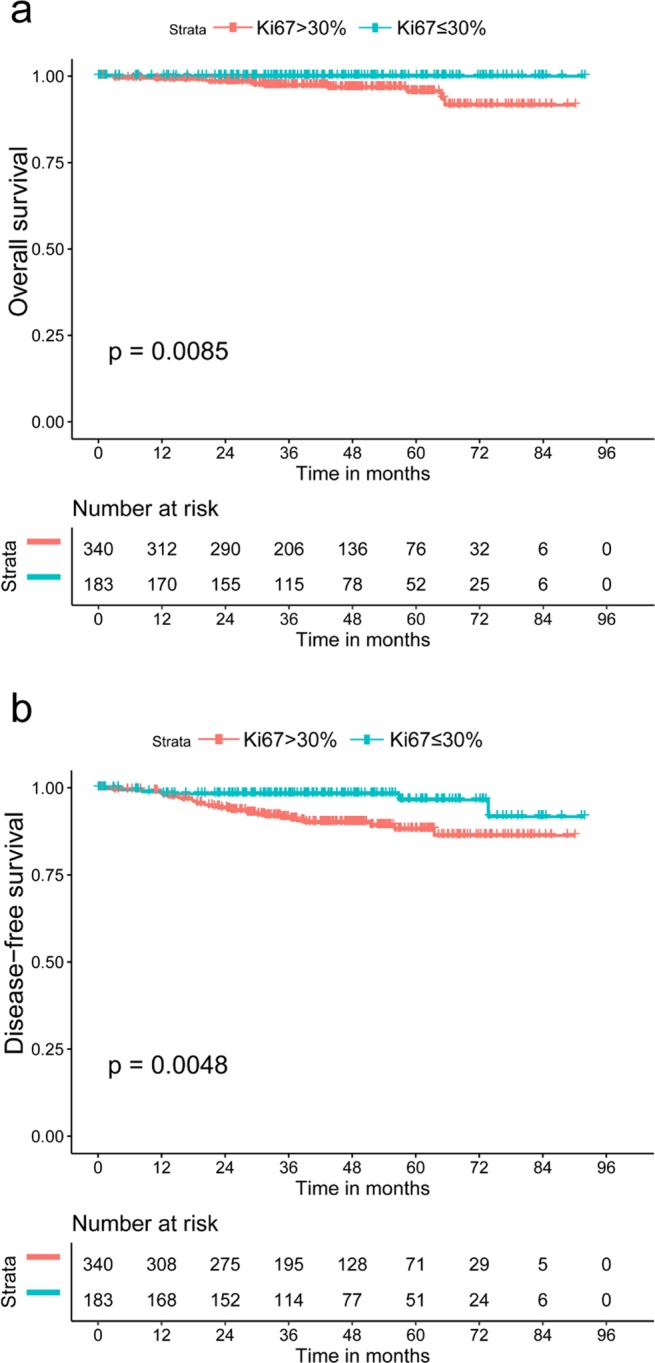
Kaplan-Meier curve by Ki-67 in T_1_N_0_M_0_ triple-negative breast cancer patients. (**a**) Overall survival; (**b**) disease-free survival.

In the subgroup analysis, the Ki-67 cutoff at 30% had prognostic and predictive potential for DFS with only either tumor size ≤2 cm (*p* = 0.008) or lymph node-negative (N−) (*p* = 0.038) in multivariate analyses compared with tumor size >2 cm (*p* = 0.275) and lymph node-positive (N+) (*p* = 0.156). For OS, in patients with tumor sizes ≤2 cm, the Ki-67^low^ group had significantly better OS (*p* = 0.017) than the Ki-67^high^ group; however, in the N- group, the Ki-67^low^ group had better OS without statistical significance (*p* = 0.077). Figure 3 shows Ki-67 as a prognostic marker for OS and DFS for the different TNBC subtypes. Considering the N-stage and T-stage comprehensively, in T1N0M0 TNBC (stage I, n = 523), the Ki-67^high^ group had significantly worse DFS (*p* = 0. 008, Table 3). Additionally, Kaplan-Meier survival analysis also confirmed that the Ki-67^high^ group was significantly associated with poorer DFS and OS, which was found in only patients with tumors ≤ 2 cm (n = 690, *p* = 0.004 and *p* = 0.005, respectively) or N- (n = 945, *p* = 0.031 and *p* = 0.046, respectively) compared with those patients with tumors >2 cm (n = 570, *p* = 0.326 and *p* = 0.664, respectively) or N+ (n = 447, *p* = 0.2, *p* = 0.46, respectively). Furthermore, in stage I TNBC, worse OS (*p* = 0.008) and DFS (*p* = 0. 005) were observed in the Ki-67^high^ group (Fig. 4a,b). In a total of 523 T_1_N_0_M_0_ TNBC patients, the DFS events in different subgroups were 0.00% (0/38) in pT_1mic_, 4.35% (1/23) in pT_1a_, 5.26% (4/76) in pT_1b_, and 8.03% (31/386) in pT_1c_.

**Table 3:** Univariate Cox regression analysis for Ki-67 as a prognostic marker for DFS and OS.

Variable	DFS			OS		
	HR	95% CI	*p* value	HR	95% CI	*p* value
**TNM**						
I	3.577	1.390–9.207	**0.008**	42.152	0.316–5628.069	0.134
II	1.270	0.756–2.133	0.366	1.300	0.647–2.612	0.461
III	0.351	0.738–2.355	0.351	1.249	0.594–2.625	0.558

Abbreviations: CI, confidence interval; HR, hazard ratio; DFS, disease-free survival; OS, overall survival. The adjusted *p* value and hazard ratio were derived from the Cox model, with Ki-67 ≤ 30% as a reference.

### Association between the expression of Ki-67 and adjuvant chemotherapy

For 945 N− TNBC patients, multivariate Cox analysis showed that adjuvant CT was an independent factor for OS in the N−/Ki-67^high^ group (*p* = 0.016), while in the N−/Ki-67^low^ group, the interaction between adjuvant CT and prognosis was not statistically significant (*p* = 0.668). Kaplan-Meier survival analysis also confirmed that adjuvant CT was associated with better OS in the N−/Ki-67^high^ group (*p* = 0.017) than in the N−/Ki-67^low^ group (*p* = 0.875) (Fig. S1a,b). The HR for OS in the N−/Ki-67^high^/without adjuvant CT group was 3.615 (*p* = 0.005, 95% CI: 1.108–3.421), which was higher than 2.408 in the N-/Ki-67^high^ group and 1.938 in the N−/without adjuvant CT group.

**Figure 5:**
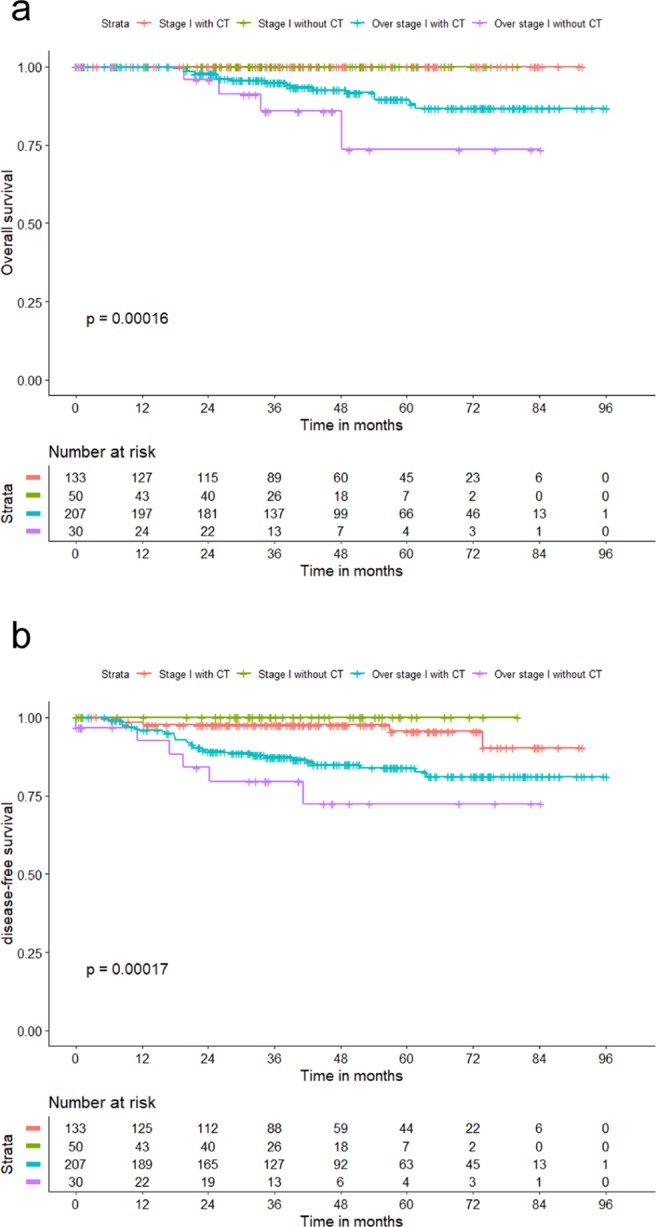
Kaplan-Meier curve by stage and adjuvant chemotherapy in Ki-67^low^ triple-negative breast cancer patients (**a**) overall survival; (**b**) disease-free survival. Abbreviations: CT, chemotherapy. Low Ki-67 expression was defined as ≤30%.

In the stage I/Ki-67^low^ group (n = 183), the median follow-up time was 39.5 months, among which five patients had recurrence without any death event. Further Kaplan-Meier analysis showed that adjuvant CT did not affect DFS (p = 0.248), while adjuvant CT played an important role in over stage I/Ki-67^low^ patients, both OS and DFS (Fig. 5a,b). For stage I patients who underwent adjuvant CT (n = 424), Ki-67 expression did not affect OS (*p* = 0.189); for those without adjuvant CT (n = 99), the Ki-67^high^ group had a higher risk of recurrence than the Ki-67^low^ group (*p* = 0.049).

## Discussion

Because of the poor prognosis of TNBC and the lack of effective treatment so far, the direction of future research involves the search for effective biomarkers to guide treatment and predict prognosis^18^. In this study, we consecutively enrolled 1800 patients to further explore the prognostic and predictive potential of Ki-67 expression in TNBC. As treated patients had more unfavorable baseline characteristics, the effect of Ki-67 expression could have been underestimated. We therefore conducted PSM in our analyses, and Cox regression analysis verified significant differences between the Ki-67^high^ group and the Ki-67^low^ group in the PSM cohort. All patients were recruited from a single clinical center, ensuring the stability of the quality of pathological marker testing, clinical diagnosis and evaluation, and treatment decisions.

Currently, since testing for Ki-67 is more convenient and economical, the assessment of the level of Ki-67 expression has been proposed in routine practice to be a measure for the quantification of cell proliferation in BC samples^19^, particularly to define prognostic subgroups of HR+ tumors. However, among most cohorts of BC studies, the number of cases of TNBC and HER2+ BC was quite small^20^, which might limit the ability of Ki-67 to predict the prognosis and identify clinically distinct subclasses. In addition to the shortcoming of a small number of samples in previous studies, a clear and uniform cutoff in TNBC has still not been established. Since baseline Ki-67 values for TNBC are much higher than those for luminal tumors^20^, the definitions of Ki-67 cutoff values in TNBC have been diverse and controversial, ranging from 10% to 61%, with one study in South Korea defining the cutoff as 10%^21^ and one study in Italy defining the cutoff as 35%^22^. In our study, we found that the optimal cutoff value of Ki-67 in TNBC patients for distinguishing prognosis was 30% in the combined PSM and multivariate Cox regression analysis. When Ki-67 expression ranged between 10 and 25%, the risk of misclassification was 37%; when Ki-67 expression was <10% or ≥25%, the risk of misclassification was only 11%^4^. We defined Ki-67 as a classification variable at a 30% threshold, which might stabilize the variability between observers and across laboratories and reduce the influence of manual interpretation errors on prognostic evaluation. Standardized cut-off values for Ki-67 have not been established, and laboratory-specific values should be used.

With Ki-67 at the 30% threshold, N-stage and T-stage were independently linked to prognosis, and those patients with Ki-67 > 30% were associated with shorter DFS and OS. However, this cutoff value had the greatest prognostic and predictive potential for prognosis in patients with a tumor size ≤ 2 cm or those who were lymph node-negative, and especially those with stage I disease. Therefore, we speculated that the Ki-67 cutoff at 30% was an early independent predictor in early invasive TNBC, but its use was limited in TNBCs over stage I. In previous studies, Ki-67 > 30% might be attributed to the early recurrence pattern of TNBC within the first three years of follow-up^23,24^. In this study, 82.4% (75/91) of relapses occurred in the first three years following the initial pathological diagnosis, and among these patients, 81.33% (61/75) had Ki-67 > 30%. Therefore, TNBC with its high proliferative potential should be followed-up more frequently in the first three years after diagnosis.

As for treatment, many studies have shown that the expression level of Ki-67 is related to the subsequent treatment selection and efficacy^25–27^. Previous studies have demonstrated that Ki-67 is one of the markers of chemosensitivity for BC, but such an association has rarely been found in TNBC. Interestingly, in this study, we found that N−/Ki-67^high^ TNBC patients could benefit from adjuvant CT; however, the advantage of CT was not clearly observed in stage I/Ki-67^low^ TNBC patients. One of the reasons behind this phenomenon might be that the low expression of Ki-67 is insensitive to CT, and another reason could be that these stage I/Ki-67^low^ patients themselves had a favorable prognosis and might be exempted from CT, which provides a basis for future de-escalation therapy. Further studies on the correlation between Ki-67 and chemosensitivity in TNBC might be necessary.

One of the limitations of our study was that there were few TNBC patients in T_1b_N_0_M_0_, and whether these patients need adjuvant CT after surgery is still controversial^28–30^; thus, we were unable to carry out in-depth survival analysis and prognosis prediction for the T_1b_N_0_M_0_ group, nor can we guide clinical practice. Besides, neoadjuvant therapy is becoming an option for more and more TNBC patients, which was not included in our study. Therefore, the application of this conclusion to neoadjuvant therapy in TNBCs is limited and deserves further study. Furthermore, Ki-67 might be even more powerful if incorporated in the design of trials of risk-adapted adjuvant therapies in the future.

## Conclusions

For TNBC patients, Ki-67 cutoff at 30% had early independent prognostic and predictive potential for OS and DFS, and Ki-67 > 30% was significantly associated with worse prognosis, especially for stage I patients. For N−/Ki-67^high^ TNBC patients, adjuvant CT may be necessary; however, for stage I/Ki-67^low^ TNBC patients, the advantage of CT is unclear, providing the basis for future de-escalation therapy. Ki-67 cutoff at 30% can be used for further classification of TNBC into two subtypes with different responses and prognoses.

## Supplementary information

Supplementary information.

## Data Availability

The data that support the findings of this study are available from the corresponding author, but restrictions apply to the availability of these data, which were used under license for the present study, and so are not publicly available. Data are, however, available from the authors upon reasonable request and with permission of the corresponding author.
