# Supplementary material for: The prognostic and predictive potential of Ki-67 in triple-negative breast cancer
Source: Sci Rep. 2020 Jan 14;10:225. doi: 10.1038/s41598-019-57094-3 (PMC6959292; doi:10.1038/s41598-019-57094-3)
Supplement: Supplementary file 1 — Supplementary information. [file 41598_2019_57094_MOESM1_ESM.pdf]

The prognostic and predictive potential of Ki-67 in triple-negative breast cancer

Xiuzhi Zhu[1,2,3#], Li Chen[1,2,3#], Binhao Huang[1,4], Yue Wang[1,5], Lei Ji [1,3], Jiong Wu[1,2,3], Genghong Di[1,2,3], Guangyu Liu[1,2,3], Ke Da Yu[1,2,3], Zhimin Shao[1,2,3,6], Zhonghua Wang[7,8,9*]

[#]Contributed equally

*Correspondence: zhonghuawang95@hotmail.com / wangzhonghua2691@sina.com.

1. Department of Oncology, Shanghai Medical College, Fudan University, 130 Dong-An Road, Shanghai, 200032, P.R. China.

2.Key Laboratory of Breast Cancer in Shanghai, Fudan University Shanghai Cancer Center, 270 Dong-An Road, Shanghai, 200032, P.R. China.

3.Department of Breast Surgery, Fudan University Shanghai Cancer Center, 270 Dong-An Road, Shanghai, 200032, P.R. China.

4. Department of Gastric Surgery, Fudan University Shanghai Cancer Center, 270 Dong-An Road, Shanghai, 200032, P.R. China.

5.Department of Pathology, Fudan University Shanghai Cancer Center, 270 Dong-An Road, Shanghai, 200032, P.R. China.

6. Institutes of Biomedical Sciences, Fudan University, Shanghai, P.R. China.

7. Department of Oncology, Shanghai Medical College, Fudan University, 130 Dong-An Road, Shanghai, 200032, P.R. China. zhonghuawang95@hotmail.com.

8.Key Laboratory of Breast Cancer in Shanghai, Fudan University Shanghai Cancer Center, 270 Dong-An Road, Shanghai, 200032, P.R. China. zhonghuawang95@hotmail.com.

9.Department of Breast Surgery, Fudan University Shanghai Cancer Center, 270 Dong-An Road, Shanghai, 200032, P.R. China. zhonghuawang95@hotmail.com/ wangzhonghua2691@sina.com.

Table S1 Characteristics of the 1392 triple-negative breast cancer patients

	After PSM n=1392		
	Ki-67[Low] （n=464）	[c]Ki-67[High] (n=928）	Total
**Variable**	Number (%)	Number (%)	
**Age（year）**		[d]p=0.4382	
≤50	155 (33.41%)	331 (35.67%)	486 (34.91%)
>50	309 (66.59%)	597 (64.33%)	906 (65.09%)
**BMI**		p=0.2918	
≤24	295 (63.58%)	569 (61.31%)	864 (62.07%)
>24	161 (34.7%)	350 (37.72%)	511 (36.71%)
Missing	8 (1.72%)	9 (0.97%)	17 (1.22%)
**Location**		p=0.6905	
Left	235 (50.65%)	482 (51.94%)	717 (51.51%)
Right	229 (49.35%)	446 (48.06%)	675 (48.49%)
**Multifocality**		p=0.5124	
No	438 (94.4%)	880 (94.83%)	1318 (94.68%)
Yes	18 (3.88%)	27 (2.91%)	45 (3.23%)
Missing	8 (1.72%)	21 (2.26%)	29 (2.08%)
**pT**		p=0.1247	
T1	233 (49.78%)	457 (49.25%)	690 (49.43%)
T2	172 (37.28%)	373 (40.19%)	545 (39.22%)
T3	13 (3.02%)	12 (1.29%)	25 (1.87%)
Missing	46 (9.91%)	86 (9.27%)	132 (9.48%)
**pN**		p=0.4767	
N0	325 (70.04%)	620 (66.81%)	945 (67.89%)
N1	88 (18.97%)	190 (20.47%)	278 (19.97%)
N2	27 (5.82%)	72 (7.76%)	99 (7.11%)
N3	24 (5.17%)	46 (4.96%)	70 (5.03%)

Abbreviations: PSM, propensity score matching.

c. High Ki-67 expression was defined as >30%.

d. p value was derived from the chi-square test

Table S2 Univariate and multivariate Cox regression analyses of factors associated with disease-free survival in triple-negative breast cancer

		Univariate			Multivariate		
**Variable**		HR	95% CI	*P* value	HR	95% CI	*P* value
**Age**	≤50 vs ＞50	1.198	0.867-1.655	0.273			
**BMI**	≤24 vs ＞24	1.272	0.935-1.731	0.126			
**Location**	Left vs right	0.898	0.664-1.215	0.486			
**Differentiation**	III vs I+II	0.991	0.696-1.4412	0.960			
**T-stage**				**0.001**			0.147
	pT2 vs pT1	1.784	1.298-2.453	0.000	1.388	0.100-1.926	0.050
	pT3 vs pT1	2.066	0.832-5.128	0.118	1.193	0.470-3.025	0.711
**Multifocality**	Yes vs no	1.252	0.553-2.837	0.590			
**N-stage**				0.000			**0.000**
	pN1 vs pN0	1.933	1.312-2.849	**0.001**	1.920	1.287-2.864	0.001
	pN2vs pN0	3.926	2.541-6.066	0.000	3.687	2.355-5.773	0.000
	pN3 vs pN0	6.451	4.196-9.917	0.000	5.521	3.461-8.806	0.000
**Ki-67**	≤30% vs ＞30%	1.591	1.126-2.249	**0.009**	1.604	1.118-2.300	**0.010**

Abbreviations: CI, confidence interval; HR, hazard ratio.

The covariates in the Cox model were all categorical variables, and the adjusted *p* value and HR were derived from the model.

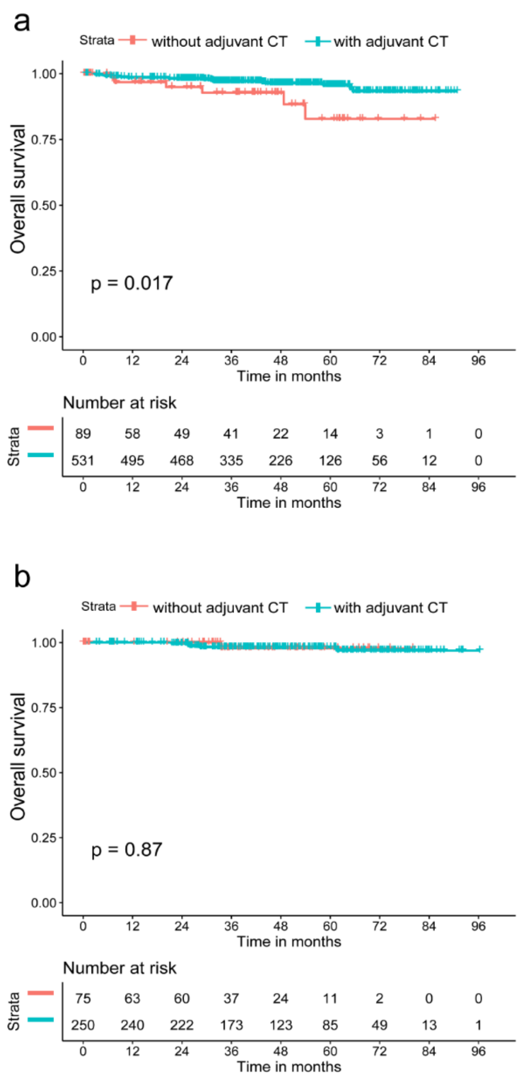

Figure S1 Kaplan-Meier curve of overall survival by adjuvant chemotherapy in lymph node-negative triple-negative breast cancer

(a) in the Ki-67^{high} group; (b) in the Ki-67^{low} group.
